# The Implementation of Evidence-Based Obesity Education Curricula to Prevent Cancer in a Predominantly Mexican–American Community on the U.S.-Mexico Border

**DOI:** 10.1007/s13187-021-02101-3

**Published:** 2021-10-08

**Authors:** Roy Valenzuela, Alma Morales, Jon Sheen, Sylvia Rangel, Jennifer J. Salinas

**Affiliations:** Center of Emphasis in Cancer, Texas Tech University Health Sciences Center, 5001 El Paso Dr El Paso, El Paso, TX 79905-2827 USA

**Keywords:** Cancer, Obesity, Mexican–American, Community-based education, Obesity prevention, Bilingual, Physical activity, Nutrition

## Abstract

Although cancer is the leading cause of death among Mexican-Americans, few community-based programs target obesity reduction as a way to reduce the prevalence of obesity-related cancer in underserved populations. Evidence suggests that obesity correlates with 13 types of cancer. The objective is to provide an overview of evaluation and selection of evidence-based content; details of the implementation process; modifications needed to tailor education programs to specific needs of different target audiences; and demonstrate challenges of implementing a community-based prevention program intended to reduce cancer incidence and mortality in Mexican-Americans. We used the Social Cognitive Theory (SCT) to develop a 10-topic menu of educational classes using elements of multiple evidence-based curricula. Outcome measures for physical activity and nutrition were determined using the International Physical Activity Questionnaire (IPAQ) and the Dietary Screener Questionnaire (DSQ). Weight status was determined using weight, body fat, and body mass index (BMI). To date, 2845 adults received wellness education from our program. Multiple delivery models were used to reach a larger audience; they included a 4-week model, 5-week model, employer model, low-income housing, 1- and 2-h sessions, and clinic encounters. Individuals were given education at multiple community locations including senior centers (14%), churches (0.6%), employers (17.6%), low-income housing (8.2%), community centers (16.6%), clinics (11.5%), and schools (32.5%). Our study indicates that our delivery model is feasible and can disseminate evidence-based obesity education. Further investigation is necessary to assess long-term behavioral change and to assess the most effective model for delivery.

## Introduction

Cancer is the leading cause of death among Mexican-Americans and larger Latinx communities [1, 2]. Reducing modifiable risk factors is essential in reducing this current reality [3]. Documentation associates obesity with thirteen types of cancers, including the colon, uterine, and liver [3], and new evidence suggests that the increasing prevalence of overweight and obesity may be primarily responsible for growing cancer incidence and mortality in Mexican-Americans [4]. Therefore, targeting obesity as a primary cancer prevention strategy may significantly reduce the cancer burden in this population.

There has been a concerted effort to reverse current trends in obesity in Mexican-Americans through prevention and intervention programs primarily to prevent cardiovascular and metabolic diseases [5–7]. Most programs focused on middle-aged females and targeted physical activity (PA), diet, and other health indicators [5–8]. While there is evidence that these programs are successful in helping participants obtain essential tools needed to have meaningful weight loss [9, 10], there are unanswered questions as to whether these programs are implementable with similar outcomes, given duration of programs are generally between 12 weeks and 24 months long [11–14].

Programs specific to Latinx populations primarily target children and their families [15, 16] with a few that target older Hispanic adults [17]. Most of the studies we reviewed were in English and Spanish, or monolingual English, and few studies were in monolingual Spanish [9, 10]. The majority of programs were conducted with Mexican descendant populations, followed by Puerto Rican, Cuban, Guatemalan, El Salvadorian, and Honduran populations [8, 14]. Border populations were not tested, despite the predominantly Mexican–American population living on the U.S.-Mexico border. Based on substantial variations in currently available evidence-based obesity prevention programs, there is no one-size-fits-all option that has worked in all settings.

Implementing programs with populations and in environments not available during validation testing is a significant challenge. Most programs utilized Non-Latinx, urban Latinx, or non-Mexican–American Latinx populations [4, 5, 9, 18]. For example, El Paso, TX, is located on the Texas-Mexico border and is populated by Mexican-Americans (82%) that are U.S. born or immigrants [19]. In other regions of the country, Latinx groups are largely immigrant or second generation, the majority of residents in El Paso are U.S.-born bilingual, English dominant [20]. Also, in contrast, Mexican-Americans living in El Paso have a higher average high school graduation rate (84.8% vs. 80%) compared to Latinx students in California and higher median incomes than the Hispanic national average ($42,307 vs. $34,000) [21].

*Pasos Para Prevenir Cancer* is a community-based primary prevention program intended to reduce cancer incidence and mortality in Mexican-Americans living in El Paso, TX. The program utilizes curricula from evidence-based programs. This paper provides an overview of the evaluation and selection of evidence-based content, details of the implementation process, modifications needed to tailor to specific needs of different target audiences, and challenges to implementation. We will also provide lessons learned and next steps in widespread uptake of the *Pasos Para Prevenir Cancer* program.

## Methods and Results

### Program Overview

*Pasos Para Prevenir Cancer* is a primary prevention program funded by the Cancer Prevention and Research Institute of Texas (CPRIT) (PP180026). The program’s purpose is to provide obesity-related cancer prevention education to adults aged 18 years and older. We conducted a comprehensive review of existing programs and used elements from multiple evidence-based curricula to create a ten topic menu of courses. The menu allowed us to select educational material that met the needs of a socioeconomically diverse Mexican–American population living in El Paso, TX.

### Theoretical Framework: Social Cognitive Theory (SCT)

We used the Social Cognitive Theory (SCT) as the underpinning of our curriculum design. Albert Bandura developed the SCT as a framework for understanding human behaviors [22]. The core premise of the theory is that humans learn and adapt behavior based on interactions with others and their environment. The three concepts that we used from the SCT were cognitive, behavioral, and environmental factors to influence behavioral change to prevent obesity. First, we provided education to improve knowledge or attitudes about obesity, nutrition, and PA using cognitive factors incorporated into the curriculum and adapted from other evidence-based programs. Second, an opportunity to improve skills and change perceived self-efficacy through cooking demonstrations or group activities aided in influencing behavioral factors that affect obesity. Finally, the program facilitated the change of social norms around food and PA, improved access to resources in the community, and provided participants tools to influence friends and family to change healthy eating and active living behaviors. Each session addressed at least one cognitive, behavioral, and environmental factor.

### Program Development Process

The implementation process consisted of multiple steps using multiple strategies to maximize uptake and maintain fidelity to the evidence-based programming as much as possible [23]. A timeline of implementation milestones is available in Table 1. Each step of the process is accompanied by immediate, long-term, and unexpected outcomes and challenges encountered at each step. Primary project steps included developing a rationale based on current epidemiological trends in obesity and cancer incidence and mortality. The next step included an exhaustive review of evidence-based nutrition, physical activity, or obesity education programs that were bilingual to assure fidelity to evidence base at the same time as effectively reaching the El Paso community. Intervention development occurred in iterations. We began with 4 sessions; however, in response to the feedback from community partners, we re-examined our approach and made modifications to improve uptake and session quality. As part of this process, we identified barriers and facilitators of program implementation and modifications were designed to address each. An unanticipated challenge to implementation was the onset of the COVID-19 pandemic during implementation. This required further tailoring that included web-based education to adhere to social distancing guidelines due to COVID-19.

**Table 1:** Pasos Para Prevenir Cancer program timeline and implementation outcomes

Year	Project steps	Immediate outcomes	Long-term outcomes	Unexpected outcomes	Challenges
2018	• Program rationale	• A third of Mexican-Americans are obese • Obesity is associated with 13 types of cancer • 82% of the population in El Paso is Mexican–American • PPPC is intended to reduce cancer and mortality in Mexican-Americans	• Cancer is the leading cause of death among Mexican-Americans • Goal is to reduce modifiable risk factors	• Effective cancer prevention programs need to focus on obesity for this population • Majority are bilingual, English dominant • Present higher school graduation rates • Higher median income	• Determining the most effective way to implement an obesity prevention program
	• Evidence-based program search	• Searched for evidence-based nutrition, PA, and obesity programs • SCT provided the framework for delivery	• Found 17 evidence-based nutrition, PA, and obesity programs • Reviewed 32 programs • Found 2 articles in Spanish and English	• Most programs incorporated women and children • Few addressed older adults and men • Majority of programs followed Latinx groups of Mexican descent, followed by Puerto Rican, Cuban, Guatemalan, El Salvadorian, and Honduran populations • Most programs targeted children and their families	• Most programs targeted Non-Hispanic monolinguals • Efficacy was determined using pretest–posttest models
	• Intervention development	• Evidence-based nutrition and physical activity program curricula • Obesity-related cancer primary prevention • Culturally sensitive to socioeconomic situations	• Created options for modification that are sensitive to different audiences • Program began with a four-session calendar covering the curriculum topics	• Program was dual language to meet the needs of the populace • Program calendar had to become flexible to maximize participation and meet audience needs	• El Paso, Tx is comprised of English-dominant Mexican-Americans • Had to adjust to the notion of culture sensitivity • All materials used had to be bilingual
2019	• Intervention implementation • Delivery modification	• The program had to accommodate several different audiences • Calendars maximized attendance • Target audiences were emphasized	• Program attendance was well received • Parent’s preferred daytime interventions • Working subjects preferred lunchtime interventions	• 4-week model • 5-week model • Employer model • Apartment Complex competitions • 1 and 2-h sessions • Clinic Encounters	• Giveaways were offered as prizes or incentives • Shorter sessions created for those who could not participate in multiple scheduled sessions
2020	• COVID-19 virtual adaptations	• Delivered flyers to offer intervention online • Used Facebook as a streaming source	• Harder to reach audiences • Implemented the use of visual and audio online platforms	• One on one interventions were successful • Facebook was a great platform to deliver PA and nutrition interventions	• Lack of attendance after two sessions • Clinics did not know how to use us as partners • Difficulty adjusting to multiple online platforms

### Evidence-Based Program Evaluation

We conducted an extensive search of evidence-based nutrition and PA education program curricula and materials provided by several governmental wellness agencies to use as the basis for *Pasos Para Prevenir Cancer*. Criteria set for programs included consideration made on El Paso residents’ socioeconomic, cultural, and health literacy needs. In El Paso, bilingual programs are essential to maximizing English-dominant Mexican-Americans’ and younger U.S.-born participants’ reach. However, many previous programs targeted only monolingual Spanish participants [9, 10]. English-language dominance was an unexpected challenge to our notion of culture sensitivity when designing programs to reach this population. We reviewed each component of the selected program materials and identified overlapping content and content that best fit our SCT framework. Table 2 displays the topics included in the Pasos Para Prevenir Cancer curriculum with SCT construct targets and behavioral objectives.

**Table 2:** Pasos Para Prevenir Cancer topics with SCT target constructs and behavioral objectives

Intervention deliverables	SCT construct targeted	Behavioral objectives
Introduction: Ice Breaker	Social norms	Initiate obesity prevention behavior through the introduction of new social norms
Pasos Para Prevenir: Obesity Related Cancer	Knowledge	Internalize obesity-related cancer knowledge
What is Obesity and are You at Risk?	Knowledge	Internalize obesity knowledge
Activity: BMI, Do you need to lose weight? Weight control tips	Knowledge, expectations	Apply BMI concepts to own situation
Choose My Plate	Knowledge	Identify the five main food group recommendations in CMP
What did I Eat and Goal Setting	Skills, practice, self-efficacy	Establish goals to reach regular CMP compliance
Skill-building Demonstration Activity. Smartphone app, my plate menu planning, serving size	Social norms, skills, practice, self-efficacy	Increase competence in use of tools to improve healthy eating and active living
Reading a Nutrition Label	Knowledge, skills	Explain the content of a nutritional label
How to Eat Healthy When there is Limited Time/Money	Knowledge, skills	Communicate at least two solutions each for time and economic barriers to eating healthy
Activity: Smart Card – Making the Commitment	Skills, practice, self-efficacy	Define “livable” changes that the participant is able to make to eating healthy
Activities: Circular Price Finding	Skills, practice, self-efficacy	Increase competence in identifying best prices for fruits and vegetables
Introduction and Classic Favorites	Knowledge, attitudes	Identify ingredients in classic favorites that may not be healthy
Ethnic Favorites and Healthier Alternatives:	Knowledge, social norms, skills, practice	Identify healthier ingredients that could be used to replace less healthy ones
Make a Classic Better	Knowledge, social norms, skills, practice	Communicate at least two dishes where unhealthy ingredients could be swapped out for more healthy ingredients
Cooking Demo	Skills, practice, self-efficacy	Create a healthier version of a classic
Recipe Book Handouts	Skills, practice, self-efficacy, social norms, influence on others	Name one dish to commit to trying before session 4
What is physical activity and why is it important?	Knowledge	Explain the benefits of physical activity to health and preventing obesity
Activity: Physical Activity Guessing Game	Skills, practice, self-efficacy	Name one moderate and one vigorous physical activity
Getting Started With PA:	Knowledge, skills, social norms, access in the community	Describe the steps to be taken to begin selected activity
Activities: Target the right heart rate – Walking group – Muscle strengthening and stretching	Social norms, access in the community, skills, practice, self-efficacy	Increase competence in engaging in one PA-related session-based activity

### Intervention Implementation

Table 3 presents an overview of the tailored delivery options in response to feedback from participants and stakeholders. The initial offering was four sessions modified from a typical 5–12 sessions in existing curricula [9, 10, 15–17]. Topics covered background information on obesity and obesity-related cancer, recommendations for diet and PA, goal setting, understanding and using nutritional information, common barriers to healthy eating and PA, making healthier versions of favorite dishes, PA types, and how to get started and stay physically active (see Table 3). Additionally, based on the number of sessions delivered, information was delivered either during the session or using a navigation packet given to participants to take with them to learn on their own.

**Table 3:** Community education curriculum delivery options

Topic	4-week sessions	5-week sessions	Housing property	1-h session	2-h session	Navigation
Overweight & Obesity-related Cancers	C	C	C	C	C	CDC Vitalsigns
BMI & Waist Circumference	C	C	A	A	A	BMI Chart
Do you need to lose weight?	C	C	A	A	A	Tape measure
Choose MyPlate™	C	C	A	B	B	USDA’s Choose MyPlate™
Self-reflections	C	C	B	B	B	Grocery shopping tips
Understanding Nutrition Labels	C	C	A	B	B	U.S FDA Food Facts flyer
How to manage a healthy diet with a busy lifestyle	C	C	A	A	A	Grocery shopping tips
Healthier Alternatives to ethnic or comfort foods	C	C	C	A	A	Healthy recipes
Go, Slow, & Whoa Foods	C	C	C	B	B	We Can! Go, Slow, & Whoa Foods guide
Benefits of PA	C	C	A	A	A	Move Your Way flyer

*Note*. A = classroom type instruction was provided; B = only a navigation packet was provided; C = both A and B were provided

### Participants

Promotoras, health educators, and research associates engaged in community outreach to promote *Pasos Para Prevenir Cancer* in El Paso. Any adult 18 years or older could participate in the program regardless of economic or insurance status, which broadened our reach. By April 2021, 2845 participants were successfully educated in-person or virtually. Participants came from senior centers, low-income housing properties, schools, community centers, and employers. We provided each location the opportunity to tailor the content to the needs of their audience. For example, the 4-session model worked well with senior centers, so we most often offered that model when marketing to that age group. However, most employers preferred less than three sessions during lunch, so we created outreach material titled “staff development” for that population. Low-income housing residents required an entirely different approach, so we partnered with property management teams to tailor the program to fit the organization’s needs and offered incentives to residents for attending.

### 4-Week Sessions

The 4-week session was our traditional program; Table 2 denotes the topics covered in each session. This format worked well at senior centers because it provides socialization and health information to help them with existing comorbidities.

### 5-Week Sessions/Employer Model

The 5-week session is the 4-week session extended an extra week. In this model, we moved some of the content from session 1 to a new session two and adjusted the subsequent sessions a week later. We also eliminated the accompanying activities to keep the class within a 1-h window. This model was used as the standard delivery for schools and employers, even though some employers stated that attendance is generally the highest when there are only one or two sessions. Parents for school-aged children preferred daytime hours while children attended school. While working, participants were more inclined to participate during lunch or as part of staff development. Employers were able to choose from our list of topics and decided how many sessions they would like to host. This approach enabled us to reach the working population, accommodated employer needs, and complemented our walking challenges.

### Low-Income Housing

The housing property population was generally young and sometimes a mix of English-dominant and Spanish-dominant speakers, which provided us with some unique challenges. The 4-week session model did not work in this context; therefore, we worked very closely with the property management team to launch a competition for the residents. We borrowed the competition idea from our walking challenges, which were very successful among younger populations.

Similarly, we made changes to the curriculum in several ways (see Table 2). We initially used a handout to deliver information on BMI and obesity-related cancer, and participants received points for knowing their BMI. Participants were oriented to the program for the “kick-off” session and engaged in a serving size activity with actual food while the instructor reviewed Choose My Plate^©^. In the second session, we incorporated the cooking demo with a discussion of nutrition labels. At the end of the session, participants were allowed to look through the recipe binder and select a recipe that they would cook in a “cook-off” the following week. In the third session, participants were given points for participating in the “cook-off” and received extra points for explaining the dish ingredients’ nutritional value. Finally, participants earned points for engaging in PA outside of class. On the final week of class, at the cook-off, the promotora or health educator determined the winner (the participant with the most overall points) and provided certificates of completion to participants who completed the full program.

### 1- and 2-H Sessions

Due to the difficulty of getting participants back for multiple sessions in individual settings, we created a 1-h and a 2-h session option. These suggested models are in Table 2. These may be more appropriate for church groups or invited sessions that are not long-term commitments.

### Clinic Encounters

Our clinic population was a challenging demographic to reach, so we tried several different approaches to get the participants back to the clinic with limited success. Therefore, we implemented brief encounters of 15 to 30 min while patients were waiting for their providers. Nursing staff directed the health educator to potential patient rooms to provide a one on one session. The health educator provided as much information as possible during that time and gave the patient a folder to take home.

### Navigation Packet

Our program utilized a navigation (factsheets and handouts) packet to help participants follow the class they attended and provided more information about each topic. For example, the CDC Vitalsigns factsheet provided brief statistics and facts associated with the 13 obesity-related cancers and information on ways that communities can improve at the local, state, and federal levels. A body mass index (BMI) chart helped participants determine if they are underweight, healthy weight, overweight, obese, or at high risk for obesity based on this height/weight scale. This supplemental information was provided to participants to assure that each had access to all aspects of the evidence-based curricula regardless of how many sessions they participated in.

### Program Participation

 From June 2019 to April 2021, we delivered 242 sessions (2845 total participants (1681 in-person and 1164 virtual). Sessions declined at the start of COVID-19, but increased after we began to offer virtual classes. Of the 242 sessions, 80 were offered virtually. Overall, the majority were single sessions (130 (53.7%)), followed by two sessions (59 (24.3%)), three sessions (33 (13.6%)), and finally four sessions (20 (8.4%)). Table 4 presents the attendance by location type for Pre-COVID-19 and COVID-19 (March 2020 to April 2021). Prior to COVID-19, Pasos Para Prevenir Cancer was delivered in senior centers (14.0%), churches (0.6%), employers (17.6%), low-income housing complexes (8.2%), community centers (16.6%) clinics (11.5%), and schools (31.5%). During the COVID-19 pandemic, the majority of sessions were delivered through schools (37.2%), followed by employers (27.1%), community centers (9.9%), our online general webinar (6.4%), food pantries (6.1%), low-income housing (5.4%), clinics (5.2%), and churches (1.8%). Seniors centers were closed during the course of the pandemic. Parents and working adults with full-time employment constituted nearly one-half of all sessions. Additionally, while we expected to work closely with churches, church parishioners were the least represented in our program.

**Table 4:** Pasos Para Prevenir Cancer attendance log by session type (June 2019–March 2020)

Intervention Session—attendance	Pre-COVID-19		COVID-19	
	Total participants	%	Total participants	%
Senior centers	235	14.0	0	0.0
Churches	10	.6	21	1.8
Employers	296	17.6	316	27.1
Low-income housing	139	8.2	63	5.4
Community centers	279	16.6	115	9.9
Clinic encounters	196	11.5	61	5.2
Schools	526	31.5	442	37.2
General webinar			75	6.4
Food pantry			71	6.1
Total participants	1681		1164	

### Outcome Evaluation

An essential part of Pasos Para Prevenir Cancer’s evaluation was to evaluate its effect on meaningful behavioral change. As such, participants were recruited into our evaluation study after attending one of our education sessions. We attempted to recruit 1660 participants since the program’s start and have successfully enrolled 272 participants into the evaluation study. Since we recruited by phone or email, unsuccessful recruitment was largely due to non-response and not refusal. Evaluation study participants were administered a questionnaire, partook in a fitness assessment, and provided anthropometric data at baseline, 6 months, and 12 months. At the time of this report, 145 participants were eligible to have completed all three waves of data collection. Of those, 74 completed 6 months and 75 completed 12 months. While the attrition rate was substantial at 51.7%, if we were able to make contact with participants at 6 months, the retention rate was 100%. Also, it should be noted that follow-up data collection began at the outset of the COVID-19 pandemic and a number of participants were hospitalized or had died as we were informed by family.

### Outcome Measures

#### Physical Activity

Physical activity was measured using self-reported PA and fitness—the *International Physical Activity Questionnaire* (IPAQ). The IPAQ is a well-established set of survey instruments developed in 2002 to assess PA engagement [24]. There are two versions (short and long) of the questionnaire, available in multiple languages. We used the long version, 27 questions, which contains five physical activity domains: Work, Transportation, Housework/House Maintenance/Caring for Family, Leisure/Recreation Time, and Time Spent Sitting. Likert scale questions determined how many days and duration of time per day participants engaged in vigorous, moderate, and light PA for at least 10 min during the last 7 days. A separate set of questions assessed the amount of time participants spent sitting or sedentary. *Cardiorespiratory fitness* was measured using the YMCA Step Test, a 3-min test used to assess heart rate recovery by having the participant step up and down a platform at a selected cadence and then measure heart rate immediately afterward [25].

#### Nutrition

Nutrition was measured using the National Cancer Institute’s (NCI) Dietary Screener Questionnaire (DSQ). The DSQ is a 26-item interviewer-administered *dietary* recall inventory that is easy to use and is available in English and Spanish. The DSQ contains 139 questions asking participants to recall their food intake for the last 12 months. The tool lists an extensive but finite list of foods and beverages and asks the participant to select the quantity range that is the closest estimate to how many times they consumed the food or beverage over the past 12 months. For food/beverage products that are more commonly consumed, such as juice or milk, the quantity ranges go from “1 time per month or less” to “6 or more times per day,” and “NEVER” is always an option. For specific food/beverage products that are less commonly consumed, such as pineapples and cabbage, the quantity ranges are “1–6 times per year” to 2 or more times per day,” and “NEVER” is also always an option. Also, some food/beverage products require more detail, such as the consumption of a “light, low-fat, or fat-free” form of the product.

#### Clinical Measures of Obesity

Anthropometric measures (waist circumference, weight, and body mass index (BMI)) helped determine participants’ weight status. We used a non-rigid measuring tape to measure waist circumference. Proper waist circumference measurement places the tape right above the participant’s hipbones and snugly and horizontally placed around the waist. The participant exhaled before the recording of final measurements [25]. *Height* was self-reported by participants. *Weight* and *body fat percentage* were measured using an Escali Glass Body Fat/Body Water Digital Bathroom Scale – 440 lbs (Part number: 1058788). *BMI* was calculated as a ratio using the English System as weight (lb) / [height (in)] 2 × 703 [26].

#### Baseline Characteristics of Evaluation Participants

To date, the evaluation study consists of 272 participants. Table 5 presents the baseline demographic characteristics of evaluation participants and U.S. Census Bureau data for El Paso County. The majority of evaluation participants were women (84.7%), Hispanic (91.3%), born in Mexico (56%), and spoke primarily Spanish (51.0%). On average, they were 50.9 years old, had 12.3 years of education, and lived in homes where the average income was approximately $7250 per year. While participants differed from the county demographics in gender, % Hispanic, nativity, language use, education, and income, they do represent the characteristics of the evidence-based programs from which PPPC was based on [9, 10, 15–17].

**Table 5:** Demographic characteristics of evaluation study participants

	*N* (mean)	% (s.d.)	El Paso (%)
Gender (*n* (%))			
Male	23	15.3	51.0
Female	127	84.7	49.0
Ethnicity (*n* (%))			
Hispanic	137	91.3	81.4
Non-Hispanic	12	8.0	18.6
Age (mean ± s.d.)	50.9	16.2	32.6
Nativity			
U.S. born	64	42.7	77.8
Mexico/other	86	57.3	22.2
Language use in home (*n* (%))			
English	35	23.5	30.3
Spanish/both	76	76.5	69.7
Education (years (mean ± s.d.)	12.3	4.0	80
Household oncome (mean + s.d.)	$7253	$21,771	$47,568

U.S. Census Bureau data obtained from: https://data.census.gov/cedsci/all?q=&g=0500000US48141

## Discussion

Cancer is the leading cause of death among Mexican-Americans [1, 2] and obesity is one of the leading modifiable risk factors for this population [27]. *Pasos Para Prevenir Cancer* is a program developed to deliver evidence-based obesity education tailored to the diverse Mexican–American population living in El Paso, TX. Considerations for implementation included accessing bilingual programming, tailored to the different levels of health literacy and interests, and varying delivery duration. Using this tailored approach, we were able to educate more than 4000 El Pasoans over 2 years.

Most of the programs we evaluated targeted obesity prevention or reduction to help prevent heart disease or diabetes. No program focused on obesity-related cancer prevention despite growing incidence and mortality [28]. Challenges in access to cancer care have been well documented for Mexican-Americans as 17.8% are uninsured [29]. Therefore, primary prevention programs like *Pasos Para Prevenir Cancer* are needed and have the potential of curbing cancer risk by educating Latinx populations at a large scale through a tailored approach.

Tailoring evidence-based programming was required to reach large numbers of participants. In El Paso, most residents are Mexican–American; however, they vary in socioeconomic status, language preferences, and health literacy. Many programs focusing on Mexican-Americans or other minority groups targeted low-income children or older adults [13, 16, 30]. Therefore, content needed to be modified to appeal to all income ranges, all age groups, and different levels of health literacy. We also provided incentives for participants of our study in the form of resistance bands and water bottles.

## Limitations

While we were able to educate in-person or online 2845 participants in our education program, more evaluation is needed to determine which aspects of the program are most effective at inciting behavioral change. Additionally, the key to implementing tailored programs is assuring that participants receive in some way all aspects of the evidence-based information. Further investigation is needed to determine the most effective form of content delivery (i.e., session vs. navigation packet) in El Paso or similar settings. An additional limitation is that we do not have long-term data on the program’s effectiveness in creating meaningful behavioral change. Therefore, the next step to our implementation is to evaluate behavioral change and assess which program components were associated with change.

Despite limitations, *Pasos Para Prevenir Cancer* demonstrates the feasibility and a potential model for disseminating evidence-based obesity education. The next steps for *Pasos Para Prevenir Cancer* include assessing long-term behavioral change 6 and 12 months after participating in the program. Additionally, assessing different aspects of evidence-based education approaches will help determine which aspects have the best results.
